# Investigation and prediction of the severity of p53 mutants using parameters from structural calculations

**DOI:** 10.1111/j.1742-4658.2009.07124.x

**Published:** 2009-08

**Authors:** Jonas Carlsson, Thierry Soussi, Bengt Persson

**Affiliations:** 1 IFM Bioinformatics, Linköping UniversitySweden; 2 Department of Oncology-Pathology, Cancer Center Karolinska (CCK), Karolinska InstitutetStockholm, Sweden; 3 Université Pierre et Marie Curie-Paris6France; 4 Department of Cell and Molecular Biology, Karolinska InstitutetStockholm, Sweden

**Keywords:** cancer, molecular modelling, mutations, p53, structural prediction

## Abstract

A method has been developed to predict the effects of mutations in the p53 cancer suppressor gene. The new method uses novel parameters combined with previously established parameters. The most important parameter is the stability measure of the mutated structure calculated using molecular modelling. For each mutant, a severity score is reported, which can be used for classification into deleterious and nondeleterious. Both structural features and sequence properties are taken into account. The method has a prediction accuracy of 77% on all mutants and 88% on breast cancer mutations affecting WAF1 promoter binding. When compared with earlier methods, using the same dataset, our method clearly performs better. As a result of the severity score calculated for every mutant, valuable knowledge can be gained regarding p53, a protein that is believed to be involved in over 50% of all human cancers.

## Introduction

Recently, several large-scale screens for genetic alterations in human cancers have been published [1,2]. The identification of novel genes associated with tumour development will provide novel insight into the biology of cancer development, but should also identify whether some of these mutated genes could be efficient targets for anticancer drug development. Analysis of these screens has led to the finding that the prevalence of missense somatic mutations is far more frequent than expected. Moreover, this observation has been complicated by the discovery that the genome of cancer cells is polluted by somatic passenger mutations (or hitchhiking mutations) that have no active role in cancer progression and are coselected by driver mutations, which are the true driving force for cell transformation [3].

Passenger mutations can be found in coding or noncoding regions of any gene, and distinguishing them from driving mutations is a difficult but necessary task in order to obtain an accurate picture of the cancer genome. Several statistical approaches have been developed to solve this problem, such as comparing the observed to expected ratios of synonymous to nonsynonymous variants. Alternatively, various bioinformatics methods can be used to provide an indication of whether an amino acid substitution is likely to damage protein function on the basis of either conservation through species or whether or not the amino acid change is conservative [4].

Predicting the effects of amino acid substitutions on protein function can be a powerful method, and several algorithms have been developed recently [4–7]. The major drawback of these analyses is the lack of information regarding the activity or loss of activity of the target protein, as only a few variants (< 100) have been fully analysed. In this regard, analysis of the p53 gene can be a paradigm for this type of analysis. First, p53 gene mutations are the most common genetic modifications found in more than 50% of human cancers [8]. Almost 80% of p53 mutations are missense mutations, leading to the synthesis of a stable protein lacking its specific DNA binding activity. The latest version of the UMD_p53 database contains 28 000 p53 mutations, corresponding to 4147 mutants that were found with a frequency ranging from once (2218 mutants) to 1264 times (one mutant, R175H) [9]. A second advantage of p53 mutation analysis, and a unique feature of this database, is the availability of the residual activity of the majority of p53 missense mutants. The biological activity of mutant p53 has been evaluated *in vitro* in a yeast system using eight different transcription promoters [10]. Third, the three-dimensional structure of the p53 core domain, where the majority of p53 mutations are located, has been solved, which allows the inclusion of structural data in a predictive algorithm. Last, phylogenetic studies of p53 have been extensive, and more than 50 sequences from p53 or p53 family members are available in various species, ranging from *Caenorhabditis elegans* and *Drosophila* to a large number of vertebrates [11].

With all this information on p53, there is an excellent opportunity for structural calculations and the development of methods to predict the severity of p53 mutations. In a recent study, we have successfully used structural calculation techniques in studies of mutants in human steroid 21-hydroxylase (CYP21A2), causing congenital adrenal hyperplasia [12]. Using structural calculations of around 60 known mutants, we managed in all cases but one to explain why specific mutations belonged to one of four different severity classes. This was accomplished by investigating several parameters, in combination with the inspection of the structural models. In the light of this achievement, we have applied a similar approach to p53 to arrive at an automated method for the prediction of mutant severity. In this paper, we show that this is possible and that we can achieve a prediction accuracy of 77%.

## Results

In this study, we have investigated correlations between human p53 mutants found in cancer patients and the corresponding activity of promoter binding. The aim was to obtain a better understanding of molecular mechanisms to explain why certain mutations cause more severe effects than others and to be able to predict the severity of new, hitherto uncharacterized mutants.

### Initial parameter investigation

**Fig. 1:**
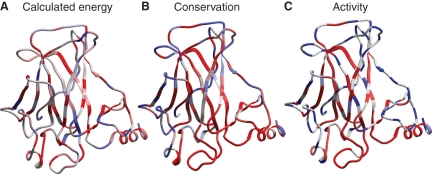
Comparison of calculated energy (A), positional conservation (B) and transactivating activity (C) of p53 mutants. The structure is based on the 1tsr crystal structure of p53. In (A), p53 is coloured according to the calculated energy for mutants at each position. Red indicates high energy and blue low energy. In (B), the colours illustrate conservation, where red corresponds to highly conserved and blue to nonconserved residues. In (C), the positions are colour coded from red to blue, where red indicates most severe and blue wild-type activity.

For the initial development of the PREDMUT method, two parameters were investigated: sequence conservation and *in silico-*calculated molecular stability for a specific mutant, which are described in more detail later. Correlations between these two parameters and impaired transactivating activity of mutants were searched for in order to identify important regions of p53. This is illustrated by projection of the properties onto the three-dimensional structure of the p53 core domain (Fig. 1). In Fig. 1A, it can be seen that positions with residue exchanges having high energy are present in every part of the protein, with a slight preference for the core β-sheet structures. In Fig. 1B, it can be seen that many of the highly conserved residues (red) are located in the core β-region, but also in the DNA binding loops. When comparing these figures, there are many similarities, but also some disagreement. Examples of disagreement are residues R156, with high energy but low conservation, and G244, with low energy but high conservation. In these cases, it is hard to determine which of the observations best correspond to reality. Figure 1C shows the experimentally determined activity, illustrating that, for R156, the energy property correlates with the activity, whereas, for G244, the conservation parameter correlates. Thus, these two parameters alone are not sufficient to make accurate predictions about the severity of a mutant, even though they contain useful information. Therefore, the PREDMUT algorithm was developed based on a much larger set of parameters.

### PREDMUT prediction algorithm

**Fig. 2:**
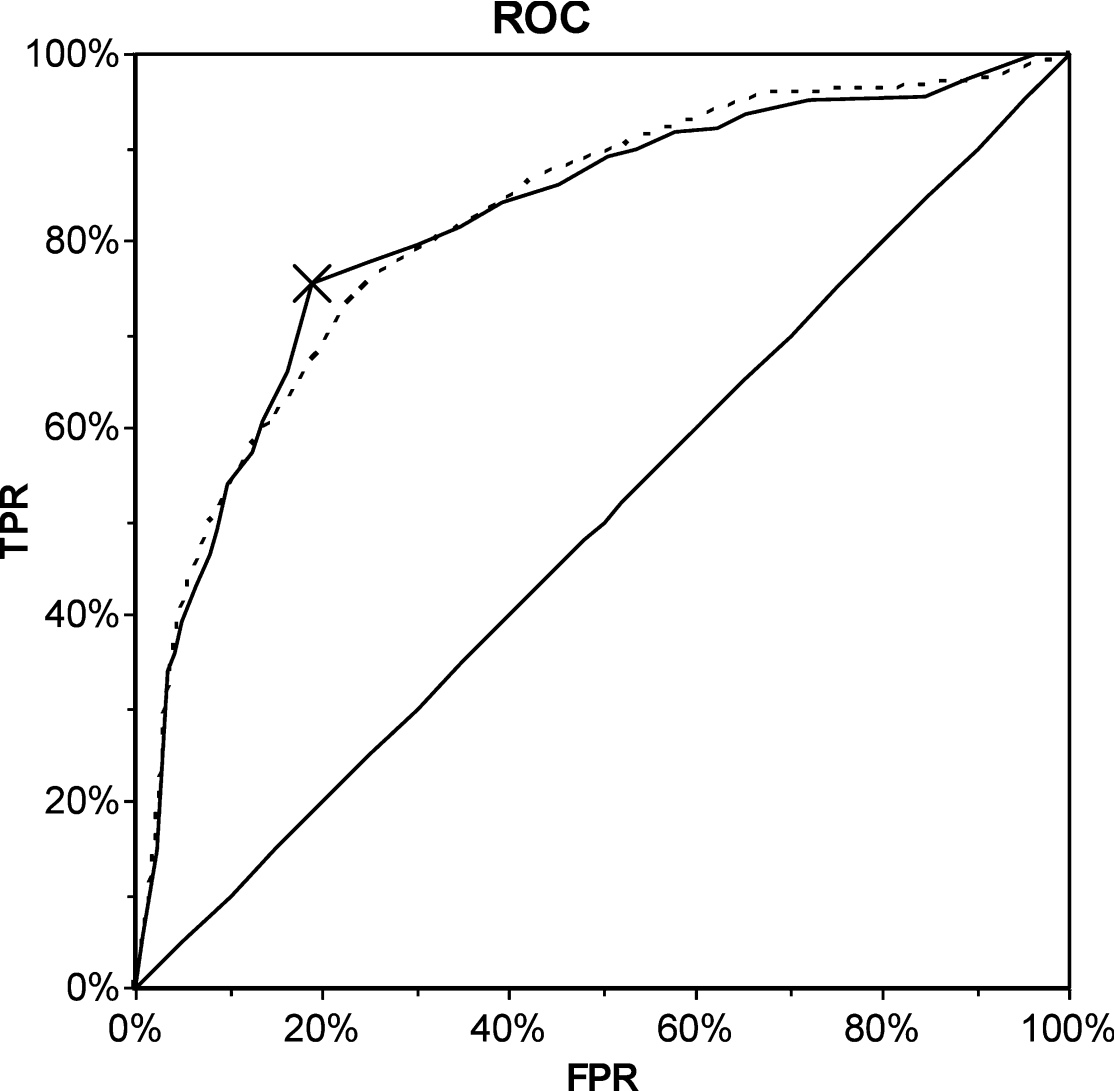
ROC curve. True positive rate (TPR) and false positive rate (FPR) depending on the cut-off value used to discriminate between the two severity classes in the test data. The broken line represents prediction on test data and the full line on training data. The straight line represents a random classification and the cross indicates the cut-off value used in PREDMUT.

The PREDMUT prediction algorithm is described in detail in Materials and methods. Using 12 different and complementary parameters (Table 1), the prediction algorithm manages to classify the training data with, on average, 79% accuracy, and to classify the test data with, on average, slightly lower than 77% accuracy and Matthews’ correlation coefficient (MCC) of 0.52. Individual results from the six controlled test runs are shown in Table 2. The total accuracy is in the range 74–81% in total, 72–85% for severe mutants and 70–79% for nonsevere mutants. The prediction power of the algorithm can also be viewed in the form of a receiver operating characteristic (ROC) curve, which is shown in Fig. 2. Here, the severity cut-off value is varied, which, when increased, raises the accuracy for severe mutations and decreases the accuracy for nonsevere mutations, and vice versa when decreased.

**Table 2:** Prediction accuracy (%) for each of the six test runs on p53 cancer mutants, where each run was trained on five-sixths of the mutants and tested on the remaining one-sixth.

Test run	Total	Class 1 (< 25% activity)	Class 2 (> 25% activity)
1	81	74	85
2	76	73	77
3	79	79	79
4	75	70	78
5	76	70	82
6	74	77	72
Total	77	74	79

**Table 1:** Description of the 12 parameters used to predict the severity of p53 mutants. Asterisks denote parameters calculated using icm.

Parameter	Explanation
Accessibility*	Percentage of amino acid residues buried inside the protein when a sphere with the size of a water molecule van der Waals’ radius is rolled over the protein surface
Similarity of the surroundings*	Measure of the percentage of amino acid residues inside a sphere of 5 Å that have the same polarity or charge as the wild-type
DNA/zinc	If the amino acid residue is, according to Martin *et al.* [38], involved in DNA or zinc binding
Pocket/cavity*	A cavity is a volume inside the protein that is not occupied by any atom from the protein and not accessible from the outside. A pocket is a cleft into the protein with volume and depth above default values in icm. For an amino acid residue to be a cavity or pocket, it must have at least one atom involved in defining the surface of the cavity or pocket
Calculated energy*	The calculated energy of the protein after residue exchange
Average calculated energy*	The average calculated energy of all 19 possible residue exchanges at a given position
Secondary structure*	If the exchanged residue is located in a regular secondary structure element, determined by the DSSP algorithm [39]
Hydrophobicity difference	Change in hydrophobicity value according to the Kyte and Doolittle scale [40]
Size difference	Change in size between native and new amino acid residue as defined in Protscale [41]
Amino acid similarity	The amino acid similarity between native and mutated residues, as classified in ClustalX [42]. ‘:’ corresponds to residues with conserved properties and has a value of 0; ‘.’ corresponds to semiconserved properties and has a value of 0.5; if no similarity exists, the parameter has a value of 1
Polarity change	If the mutant causes polarity or charge changes. Change equals unity and no change equals zero
Conservation	Percentage conservation at each position using p53 homologues of the vertebrate subphylum. The species included are listed in Table S1.

We also tested the algorithm on a subset of breast cancer-specific mutations with a prediction accuracy of 88% (Table S2). Only mutants with an observed frequency over five in cancer were included in this dataset, resulting in 342 mutations. The nonsevere mutations are classified correctly in 85% of cases and the severe mutations in 89% of cases, giving an MCC value of 0.66. If mutations are sorted according to frequency, the 49 most frequent mutations are predicted correctly. For the 12% that are not correctly classified, we found some common properties. Among the 31 wrongly predicted severe mutations, 20 correspond to residue side-chains exposed to the surface (65% versus 13% for correctly predicted mutations) and 17 correspond to residue exchange with similar properties (55% versus 24%). Together, these two properties explain why 29 of the 31 wrongly predicted mutations are hard to predict. Among the nine wrongly predicted nonsevere mutations, two are DNA/zinc binding (22% versus 0%) and six are completely conserved (67% versus 15%). Together, this explains the difficulty in predicting seven of the nine wrongly classified nonsevere mutations.

### 25% activity delineates severe and nonsevere mutants

The limit between the classes was set to the activity value of 25%, because this value was observed to be a natural divider of the data. The algorithm was also evaluated with other separation limits between the classes (1%, 2%, 3%, 5%, 10%, 15%, 20%, 30% and 40% activity) but, in all of these cases except for the 1% value, the data were always harder to separate (see Table 3). In the case of the 1% limit, the distribution between the two classes is highly skewed. A prediction stating that all mutations were nonsevere would result in 89% prediction accuracy. However, the MCC of such a prediction is zero. Thus, the 25% value seems to be an optimal class divider.

**Table 3:** Effect of cut-off value on the prediction accuracy. The prediction accuracy, specificity, sensitivity, number of mutants classified and MCC values on training data using different activity thresholds to delineate between severe and nonsevere mutants.

		Class 1			Class 2			
Activity cut-off value (%)	Prediction accuracy (%)	Specificity (%)	Sensitivity (%)	Number of mutants	Specificity (%)	Sensitivity (%)	Number of mutants	MCC
1	78.9	73.1	31.5	130	79.7	95.9	1018	0.38
2	78.4	76.1	35.9	155	78.8	95.5	993	0.42
3	76.1	78.4	36.2	172	75.7	95.2	976	0.41
5	73.9	81.5	39.1	206	72.2	94.7	942	0.43
10	72.3	83.4	51.7	336	67.7	90.8	812	0.47
15	78.1	79.6	75.1	541	76.5	80.8	607	0.56
20	78.4	79.3	80.8	642	77.5	75.9	524	0.57
25	78.7	81.0	82.3	669	75.6	74.1	479	0.57
30	77.8	78.0	84.6	706	77.4	68.8	442	0.54
40	76.9	75.2	88.8	773	80.5	61.2	375	0.53

**Fig. 3:**
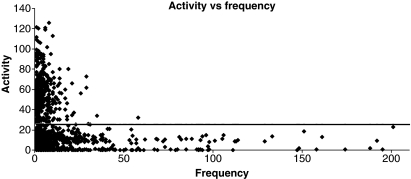
Activity versus frequency. The WAF1 activity of p53 mutations is plotted against the number of times they are found in human cancer patients. The most frequent mutations, the hotspot mutations, are not included. However, they all have activity below 25%.

Biological support of the 25% activity limit can be found by looking at the frequency distribution of the mutations. Mutations found with high frequency in humans should also be those that cause cancer, whereas the low-frequency mutations often are passenger mutations. As can be observed in Fig. 3, almost all of the high-frequency mutations have an average activity of less than 25%. In total, there are 15 272 mutations found with lower than 25% activity and only 888 mutations found with over 25% activity. This corresponds to an average mutation frequency of 47 versus 8. In addition, the average frequency of mutations with 20–25% activity is still high, with a value of 24, whereas the frequency decreases to 13 for mutants with 25–30% activity.

### Parameter weights

The different parameter weights in the prediction algorithm can provide crucial information. In Table 4, the parameters and their corresponding weights are listed for the WAF1 promoter. As WAF1 has well-defined binding characteristics [13], it was chosen as the first promoter for the development of PREDMUT. The parameters are divided into three classes: general property, position specific and mutant specific. The general property class contains parameters that are protein independent, but mutant dependent. The position-specific class includes parameters that are protein dependent, but does not reflect the resulting amino acid residue after mutation. Finally, the mutant-specific class, including only one parameter, contains information dependent on both protein and mutant.

**Table 4:** Parameter weights calculated by PREDMUT and PLS for the WAF1 promoter, together with parameter classification. General property parameters are completely protein nonspecific, position-specific parameters are dependent on the position in the protein and mutant-specific parameters depend on the position and type of amino acid residue substitution.

Parameter	Weight PREDMUT	Weight PLS	Class
Accessibility	22	20	Position specific
Conservation	16	24	Position specific
Average calculated energy	13	14	Position specific
Size change	12	6	General property
Calculated energy	11	8	Mutant specific
Similar amino acids	8	9	General property
Hydrophobicity difference	−7	3	General property
Secondary structure	−4	−1	Position specific
Polarity change	−2	0	General property
Pocket/cavity	2	−6	Position specific
Surrounding amino acids	−1	−1	Position specific

Not surprisingly, conservation is found to be a very important factor for the severity of a mutant. Accessibility is also shown to be important; this is natural as side-chains at the surface possess fewer spatial restraints and are thereby less often correlated with severe mutations. Other intuitively important factors are the similar amino acid variable and size change variable, as large changes in property and size of an amino acid residue could affect the protein negatively.

**Fig. 4:**
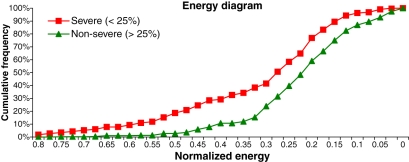
Energy diagram. Cumulative frequency of severe and nonsevere mutants, respectively, plotted against the normalized average calculated energy for all mutants.

The novel variables, the calculated energy for a specific residue exchange and for the average of all amino acid substitutions at one position, are the third and fourth (see Table 5A) most important variables, respectively. The combined weight of the two energy variables is even larger than the individual weights for both conservation and accessibility (see Table 5B), making it possible to increase the prediction accuracy compared with earlier prediction algorithms. In Fig. 4, the energy parameter is studied in more detail. Here, all mutants of the two classes are ranked according to their average calculated energy. The diagram shows decreasing energy on the *x*-axis, and the number of mutations with this or higher energy on the *y*-axis. For severe mutants, the number of mutants increases at high energy values, causing a gap between the curves representing severe and nonsevere mutants. The separation is not complete between the two classes, but there is a clear difference. One can, for example, observe that, if a mutant has a normalized energy of 0.5 or more, it is extremely likely to be a severe mutant, as only 2.7% of the nonsevere mutants possess such high energy compared with 18.6% of severe mutants, or a 1 : 7 ratio. If we look at the energy value 0.325, we still have a ratio of 1 : 2.5, or 71% probability in favour of a severe mutant. At the other end of the spectrum, where we have low energy, there is 75% probability for the mutation to be nonsevere if the energy is 0.125 or lower. Thus, on the basis of this variable alone, we can make reasonably accurate predictions on 35% of the severe mutations and on 20% of the nonsevere mutations. Even in the most difficult case, an energy value of 0.225, the variable provides useful information, as we have a prediction accuracy of 58%. This result is similar to those in earlier studies on steroid 21-hydroxylase, CYP21A2 [12]. The calculated energy is the only parameter that is specific to both position in the protein and the type of residue exchange. This adds valuable information when discriminating between two similar mutations at different positions in the protein.

**Table 5:** Parameter weights for all promoters. (A) Average and individual weights for all parameters for each promoter. Values are sorted in descending order according to the absolute value of the average weight. (B) Average and individual weights for the grouped parameters for each promoter. Values are sorted in descending order according to the absolute value of the average weight. Parameters that are similar are grouped together. Energy = Energy of mutant + Average energy of mutant. General properties = Similar amino acids + Size change + Hydrophobicity difference + Polarity change. Other = Surrounding amino acids + Two-dimensional structure + Pocket/cavity.

Parameter	WAF1	MDM2	BAX	14-3-3-σ	AIP	GAD45	NOXA	p53R2	Average
**A**									
Conservation	16	24	25	30	27	21	15	21	22
Accessibility	22	15	7	14	16	27	31	43	22
Average calculated energy	13	10	28	14	24	25	12	11	17
Calculated energy	11	10	14	18	8	8	23	0	11
Similar amino acids	8	7	6	9	4	3	6	8	7
Size change	12	15	−1	−5	9	−3	5	1	6
Hydrophobicity difference	−7	−10	2	7	0	3	0	7	4
Surrounding amino acids	−1	5	10	−1	6	−2	−6	−1	4
Two-dimensional structure	−4	2	−2	−1	2	3	2	−2	2
Polarity change	−2	0	2	2	4	3	0	−4	2
Pocket/cavity	2	2	2	0	1	1	0	2	1
**B**									
Energy	24	20	42	32	32	33	35	11	29
Conservation	16	24	25	30	27	21	15	21	22
Accessibility	22	15	7	14	16	27	31	43	22
General properties	30	32	11	23	16	13	11	20	19
Other	7	10	15	2	9	6	8	5	8

The weights for the parameters extracted from the partial least-squares (PLS) method (Table 4) show good agreement with those for our PREDMUT method: the six most important parameters are the same, with a total weight of 82% for our method and 81% for the PLS method.

Analogous to the prediction of the WAF1 promoter, we developed prediction schemes for seven other promoters (MDM2, BAX, 14-3-3-σ, AIP, GAD45, NOXA, p53R2). These classifications were shown to perform with similar prediction scores (Table 6).

**Table 6:** Promoter prediction results (%) for eight p53-related promoters.

Promoter	Training set	Test set
WAF1	79	77
MDM2	76	72
BAX	77	74
14-3-3-σ	77	74
AIP	78	75
GAD45	80	74
NOXA	80	75
p53R2	80	75

The parameter weights used in the predictions of all eight promoters are shown in Table 5A. Every column sums to 100, using absolute values, so the weights are directly comparable. The DNA/zinc parameter is not included in the table as its weight, for technical reasons, was limited to few values in the algorithm, and it only contains information for a few mutants.

In Table 5B, similar properties are grouped together. The weights are added using absolute values in order to be able to judge the importance of all parameters, regardless of their signs. We see that the energy parameter is, on average, responsible for almost one-third of the information used in the prediction. Conservation, which is commonly used in predictions, and accessibility contain almost one-quarter each of the information, which is only slightly more information than can be gathered from just looking at the general properties of the residue replacement.

The weights are generally stable, with mutual parameter rankings possessing only a few swaps in position. This indicates that the algorithm provides a classification that is optimal or at least close to optimal using linear separation.

The differences in weight for the promoters could be interpreted as reflecting differences in the mode of binding. The promoter p53R2 seems to be less dependent on the stability of the protein, indicating that it either possesses more relaxed binding that tolerates small changes in structure, or that it binds harder and thereby stabilizes the protein. BAX, however, seems to be very sensitive to structural changes.

### Cross-correlation between parameters

When applying the Pearson product-moment correlation coefficient [14] on all possible pairs of parameters, we can see that a few of the parameters show some correlation. In Table 7, we highlight the parameters with the highest correlation. The two energy parameters are partly correlated, as are conservation and accessibility, and secondary structure and accessibility. The four parameters that reflect amino acid properties are also correlated. This explains how the hydrophobicity difference can be negative for some promoters, as it is the total weight (as shown in Table 5B) of these four parameters that best describe this phenomenon. However, when testing to remove any of the parameters, the prediction became slightly worse, showing that all parameters are necessary and that they complement each other.

**Table 7:** Cross-correlation between parameters. Parameters that show the highest pairwise correlation coefficients are shown. All other correlation coefficients are below 0.3, with the majority below 0.1.

Parameter	Calculated energy		
Average calculated energy	0.48		
Parameter	Conservation	Two-dimensional structure	
Accessibility	0.45	0.46	
Parameter	Hydrophobicity difference	Similarity change	Size change
Polarity change	0.43	0.47	0.25
Hydrophobicity difference		0.63	0.34
Similarity change			0.43

### Other classification techniques

Other classification techniques were investigated to evaluate whether they could add improvements to the new method. To further investigate differences between the two classes, the data were analysed using principal component analysis in SIMCA-P 11 [15,16]. However, the data could only be partially separated when considering the first two components. Thus, using only principal component analysis on the data is not sufficiently powerful to provide an accurate prediction. Another popular method for classification is support vector machines (SVMs) [17], and several kernels [radial, dot, sigmoid and polynomial (using values of two to six as the polynomial)] were tested using the SVM implementation in icm. The best SVM used the polynomial kernel with a value of five as the polynomial (see Table 8). The total prediction accuracy is similar to that of PREDMUT. However, the weights for the individual parameters are not known, making it impossible to determine the contributions of each parameter to the final classification.

**Table 8:** Prediction accuracy (%) for the best of the methods tested and their respective MCC values.

Prediction method	Total prediction accuracy	Class 1 (< 25% activity)	Class 2 (> 25% activity)	MCC
SVM (p = 5)	76.7	82.5	68.6	0.52
PLS	73.3	86.7	63.0	0.50
PREDMUT	76.6	73.7	78.7	0.52

Furthermore, PLS was investigated using SIMCA-P 11 [16]. This method performed with slightly lower prediction quality than PREDMUT. In addition, the nonsevere classification of only 63% is on the low side and the MCC value of 0.50 is slightly lower than that of PREDMUT (see Table 8).

### Cut-off safety margin

Sometimes, when the algorithm decides whether or not a mutation is severe, the severity score is very close to the cut-off, making the prediction of that particular mutant uncertain. By introducing a small safety margin around the cut-off value, the prediction results outside this margin can be improved. The mutants that possess a score within the safety margin are classified as having unknown severity. In Table 9, the prediction accuracy is shown using difference sizes of the safety margin. By increasing the safety margin, we can go from 77% accuracy and an MCC value of 0.52 to 88% accuracy and an MCC value of 0.74. The drawback is that, in the latter case, only 45% of the mutants are classified.

**Table 9:** Prediction accuracy (%) depending on the size of the safety margin (%) used around the cut-off value. Mutants with a severity score inside the safety margin were classified as unknown.

Safety margin	Total prediction accuracy	Class 1 (< 25% activity)	Class 2 (> 25% activity)	Unknown	MCC
0	76.6	78.7	73.6	0	0.52
5	78.3	80.4	75.3	11.4	0.55
10	80.2	83.4	75.8	23.3	0.59
15	82.6	85.6	78.3	34.9	0.64
20	85.5	89.1	80.5	46.0	0.70
25	87.6	91.1	82.6	54.9	0.74

### Hotspot mutants

There are several p53 mutants that are extremely over-represented in human cancers, for example three lung cancer mutants induced by smoking described by Denissenko *et al.* [18]. It was therefore interesting to investigate how these mutants score using our prediction algorithm. In the case of R273C, R273H, R248W and R248Q, they are fairly easy to predict as they are involved in DNA binding. However, if the information about DNA binding is removed, all but R248Q are still correctly classified, mostly depending on their high conservation, but the high energy and low accessibility are also important factors. Looking at nonDNA binders, R175H, G245S, R249S and R282W, they are also highly conserved, but here the high energy and low accessibility of the mutants contribute equally to the total severity score. The above examples of eight frequent mutants are all correctly predicted with the new method. Indeed, the prediction accuracy greatly increases with mutation frequency, even though this information is not included in the data. The low-frequency mutants (frequency below six) have a 75% prediction accuracy on the training data, whereas the high-frequency mutants have 84% prediction accuracy. If the frequency cut-off is further increased to 10, the accuracy increases to 88%, 95% at frequency 40, and 100% at frequency 80. Thus, all very frequent mutants are correctly predicted using PREDMUT.

### Thermally sensitive mutants

In contrast with initial beliefs, thermally sensitive mutants were only slightly harder to predict than the others, with 76% correctly predicted. To be able to discriminate this type of mutant from the rest, we looked for special characteristics that were common for most of these mutants. The only overall difference found was an increased number of changes in polarity (51% versus 23%). Mutants that have a polarity change are correctly classified in 91% of cases, and so these are very easy to spot. The remaining mutants are harder to predict (60% correct), and thus require further experimental tests in order to explain their behaviour.

### Web server

A web server has been developed with the purpose of displaying information about p53 mutations. It shows information on molecular properties for all single-nucleotide mutations affecting the central domain of p53. For each variant, the values of all parameters used in the severity prediction are given. On the basis of these values, a severity score is presented, in addition to a class prediction and the activity values from Kato *et al.* [10]. Furthermore, the protein structure is shown as an interactive three-dimensional display based on the KiNG 3D viewer [19]. The amino acid residue exchanged is highlighted in red. In the interactive view, it is possible to zoom, rotate, change colours, save viewpoints, and so on. The server is available via http://www.ifm.liu.se/bioinfo under ‘Services’.

## Discussion

### Parameters

The prediction method described uses 12 parameters, each assigned a weight, reflecting the contribution of that parameter. The parameter representing the individual molecular free energy has a relatively large weight and gives a direct indication of the severity of a mutant. This is also the only parameter that is completely specific to a given mutant. The average calculated energy at each position could be interpreted as a measure of the structural robustness. If this measure is mapped onto the three-dimensional structure, structurally important regions can be discerned that could not be found by considering conservation alone. This can be useful in further studies of proteins with known three-dimensional structures, when evaluating new mutants or designing mutants in a protein that should not affect the stability of the protein. It might also be used to understand protein folding mechanisms. In Table 4, the parameters were categorized into general, position specific and mutant specific. Almost three-quarters of the information content originates from position-specific and mutant-specific parameters, showing that the structural context is very important.

### Comparison with earlier prediction methods

The prediction of the severity of p53 mutants has been attempted several times before. A direct comparison is difficult to make as different mutation datasets have been used. Many have (as have we) focused on the mutation dataset of Kato *et al.* [10]. However, different filtering and limitations to this dataset have been applied.

As we use structural information, we can only predict 1148 (codons 95–288) of 2314 (codons 2–393) mutations. However, without any filtering, our method has an MCC value of 0.52 and an accuracy of 77%.

In Align-GVGD [6,20], the mutations in which the promoters behaved differently were filtered out. In addition, a different activity cut-off of 45% was used versus 25% in our study. In this way, nonfunctional and functional mutations were predicted with 64.6% and 95% prediction accuracy, equalling an MCC value of 0.57 for 1514 mutants. If the same filtering is used on the 1148 mutations with structural information, we obtain 652 mutants and an MCC value as high as 0.64 (86% for nonfunctional and 79% for functional). When SIFT [4,5] was compared with Align-GVGD by Mathe *et al.* [20], it performed slightly worse (MCC = 0.47), whereas Dayhoff’s classification [21] made inferior predictions (MCC = 0.19).

To determine how effective our structural parameters are at predicting mutation severity, we compared them with CUPSAT [22]. By choosing the optimal cut-off value of −0.37 kcal·mol^−1^ for stability changes, CUPSAT managed to obtain an MCC value of 0.19, with slightly higher prediction accuracy for nonsevere mutations. In the same way, we chose optimal cut-off values of 0.35 and 0.30 for the two energy parameters used in PREDMUT: the average calculated energy and the calculated energy for a specific mutation. With these cut-off values, we obtained MCC values of 0.26 and 0.18. The parameters have high prediction accuracy on nonsevere mutations, making them a valuable complement to conservation analysis which performs well when predicting severe mutations. A 25% delineation between classes is used in this comparison, whereas, if 45% is used to delineate the classes, as in Mathe *et al.* [20], the results are slightly worse for both methods (MCC values of 0.16 for CUPSAT and 0.23 and 0.18 for the respective PREDMUT energy parameters).

### Interpretation of mutant severity

From the prediction algorithm, each mutant is given a severity score. This total score carries information on how much the mutant affects the activity of the protein. Further information can be gathered by considering which parameters have the largest contribution to the total score. If the most strongly contributing parameters are predominantly structurally related, the low activity probably is caused by a destabilization of the protein, whereas, if most contributions come from functionally related parameters, residues critical for the function can be expected.

An example of a structurally related mutant is one with low energy and large changes in amino acid properties, whereas a functionally related mutant could be one with rather high energy that is conserved and surface exposed. Which of the prediction parameters belongs to which group is not easily distinguished; instead, the complete picture is needed to make a correct prediction.

### Correlation between severity and frequency

The mutants show a clear correlation between severity and frequency for most of the parameters. If the high-frequency half of the mutants is compared with the low-frequency half, the high-frequency mutants are found to be more conserved (95% versus 87%), to have more deeply buried residues (84% versus 75%), to more often be DNA/zinc binders (25% versus 9%), to have higher normalized energy (0.36 versus 0.26). and so on. From this, it can be concluded that the more frequent is a mutant, the more severe it is, which is confirmed by the difference in average activity between the two groups (7.9% versus 23.7%). Therefore, it can be assumed that the less frequent mutants need some additional trigger or factor to be able to cause human cancer, whereas the high-frequency mutants can cause cancer by themselves. Thus, the consequence is that the severe mutants appear more frequently in cancer patients, whereas the nonsevere mutants may exist in similar quantity but are not found as frequently as they do not cause cancer.

In addition, there are relatively few mutants with only a small decrease in p53 activity found in cancer. From the p53 mutation database [9], it can be seen that the average number of cancer patients having a certain p53 mutation with a corresponding activity of over 50% is only 5.7, whereas it is as high as 40 on average for mutations with a corresponding activity of below 50%. This indicates that, in general, cancer-causing p53 mutations are associated with low activity.

### Infrequent and high-activity mutations

In the p53 mutation database, there are few mutations with high activity and also some mutations found only once. Some of these mutations may not be causative agents of cancer, but may only be found in cancer patients by coincidence. As cancer is such a common disease, there are bound to be some patients having a p53 mutation that has nothing to do with the cause of their cancer. Alternatively, the effect of the mutation alone is not sufficient to cause cancer without additional help from other factors. These aspects are important to bear in mind when considering p53-specific treatments.

### Difference in promoter binding

For most of the mutants, the promoters behave in similar ways, although WAF1 and MDM2 seem to be slightly more sensitive to mutations and NOXA and p53R2 slightly less so. This is indicated by the average activity of mutants in the central domain of 26% for WAF1 and 34% for MDM2, 71% for NOXA and 61% for p53R2, and around 45% for the other four promoters. For some specific mutants, the differences in activity are very large (Table 10). These mutants are therefore expected to be involved in the binding of the promoters. If the activity is comparatively low, the residue exchanges should be of special importance for the specific promoters. If the activity is comparatively high, it can be concluded that this promoter does not bind to this amino acid residue, at least not in the same way as the others. From Table 10, it can be seen that p53R2 possesses a few mutants that behave differently from the rest of the promoters. Of these, amino acid residues 243 and 275 are involved in DNA binding and 244 and 246 are in very close proximity to DNA binding. This indicates that p53R2 either does not use these residues for binding or that they are not necessary for binding as the DNA binds sufficiently hard to the other DNA binding residues. For the WAF1 and MDM2 promoters, the situation is opposite with extra high sensitivity towards certain mutants. Of these, only residue 283 is involved in DNA binding. However, residues 272 and 276 are close to DNA binding. The other four residues are further away, but at the same side of the protein, indicating a possible additional binding site needed for the WAF1 promoter.

**Table 10:** Mutants with very different behaviour depending on which promoter is measured. The top half shows mutants in which the activity for the p53R2 and NOXA promoters is similar to that of the wild-type, whereas the activity for all the other promoters measured is almost zero. The bottom half shows mutants that affect WAF1 and MDM2 more severely than the other promoters.

Mutant	Promoter	Activity (%)	Activity for the other promoters (%)
M243T	p53R2/NOXA	82–128	0–27
G244D	p53R2	131	0–2
M246I	p53R2	143	0–2
M246L	p53R2	97	0–1
M246V	p53R2	56	0–1
C275S	p53R2	223	0–1
Q192R	WAF1	32	67–135
D208E	WAF1/MDM2	2–12	36–96
T256A	WAF1	11	40–86
N263D	WAF1/MDM2	1–18	54–108
V272A	WAF1/MDM2	1–3	32–49
A276T	WAF1/MDM2	2–20	53–221
R283C	MDM2	0	25–153

### Prediction of the severity of mutants in other proteins

All parameters used for the predictions of p53 could be used for any protein with known structure. However, without sufficient training data, an automated prediction is not possible. Nevertheless, if the same weights are used as for p53, possibly somewhat tuned according to biological knowledge of the protein in question, a relative score between different mutants can be produced. This has been tested on human steroid 11-β-hydroxylase with good results (J. Carlsson, A. Wedell and B. Persson, unpublished results). The initial investigation of a number of additional proteins adds further evidence that the method generalizes well on other, nonrelated proteins.

If the purpose is to find residue exchanges that do not impair stability, the individual severity scores can be calculated for several candidate mutants and, subsequently, the mutant(s) with the lowest score(s) can be selected. If, on the other hand, the aim is a prediction of the activity, the scores are less valuable. However, if the mutants are split into three groups with the lowest scores in one group indicating wild-type activity, the intermediate scores in a middle group and the high-severity scores and thereby low activity in a third group, the mutants placed in the first and third groups can be expected to correlate well with high and low activity, respectively, if the intermediate group is sufficiently large. For the conservation score, it is important to base this on a good multiple sequence alignment with many sequences. All other parameters are independent of such ‘environmental’ effects.

## Materials and methods

### p53 activity data

Activity data are available for all single-nucleotide mutants with eight different promoters (WAF1, MDM2, BAX, 14-3-3-σ, AIP, GAD45, NOXA, and p53R2) and were taken from the work by Kato *et al.* [10], where 2314 p53 mutants were expressed (on average, 5.9 mutants per residue) and their activity measured. Data are available from the p53 website (http://p53.free.fr/Database/p53_download_db.html). Among the 2314 mutants, 1148 were localized in the central core domain of the protein and were used for training and evaluation of our prediction algorithm. Of the eight promoters, we studied the WAF1 promoter in greatest detail with additional testing and usage of different training methods. We also developed similar prediction schemes for the remaining promoters and evaluated them in the same way as for WAF1.

### Training and testing sets

The mutants were divided into two classes. Mutants with an activity above 25% were considered to be less severe and were denoted class 1 mutants (524 mutants), whereas those with lower activity were considered to be severe and were denoted class 2 (624 mutants).

To evaluate the performance of the algorithm, test sets were created. We used five-sixths of the data for training and the remaining one-sixth for evaluation. This was performed for all six combinations. Data were sorted according to activity and then evenly distributed into six representative test groups by letting the first mutant go into the first training set, the second mutation into the second training set, and so on.

### Development of the prediction method PREDMUT

A two-state classification algorithm, called PREDMUT (PREDict MUTants), was developed for prediction of the severity of p53 mutants. The method was trained on a set of known mutants and subsequently evaluated on another set.

The method is based on parameters reflecting the biochemical and structural properties of the amino acid residues affected by the mutations. In total, 12 different and complementary parameters were considered, as detailed in Table 1. A test of systematically removing one parameter at a time resulted in impaired prediction accuracy in all cases. As a preprocessing step, input data for each parameter were normalized to a value ranging from zero to unity.

In the prediction method, each of the 12 parameters was assigned a weight. These weights were optimized during the training phase (see below). To obtain the severity score for a specific mutant, the values for each of the 12 parameters were multiplied by the respective parameter weight and then summed. If this score was above a threshold calculated by the algorithm, the mutant was predicted to be severe and thereby belonging to class 2 and, if it was below, it was predicted to be nonsevere and thereby belonging to class 1.

During training of PREDMUT, a Monte Carlo technique was employed to optimize the parameters used in the prediction [24]. Initially, weights of zero were assigned to all parameters. Subsequently, an iterative process was undertaken in which a parameter weight was increased or decreased randomly by a fixed value. After each random change, the parameter settings were evaluated. If the score was improved, the parameters were retained; if the score was impaired, the recent change was rejected. However, if no improvements were found after a predefined number of iterations, one of the parameters causing impairment was randomly changed in order to determine the global optimum.

As there are many random steps involved, the algorithm can traverse the multidimensional parameter landscape in an infinite number of ways, at least in a practical sense. The predictions were improved by performing multiple training runs and, subsequently, by selecting the run that resulted in the best prediction on the training data. However, often several runs resulted in a similar set of parameter weights and thresholds, indicating a stable solution which is likely to correspond to the optimal. If infinite loops of increments and decrements of the same parameter without improvement were detected, the algorithm made a random change of another parameter in order to circumvent the problem.

When evaluating the PREDMUT algorithm, the goal was to arrive at as accurate a prediction as possible without being biased towards the larger class 2. This was obtained by minimizing the sum of the individual prediction error percentage for the two classes.

### Structural modelling and energy calculations

The three-dimensional structure of p53 was taken from the PDB entry 1tsr chain 1 [25] in the RCSB protein data bank (http://www.pdb.org), as a basis for all measurements and simulations. For each mutant, the residue exchange was inferred to the structure, whereafter its energy was minimized using the Monte Carlo method implemented in icm [26,27], similar to that described for CYP21A2 [12].

The structure was repeatedly minimized, alternating between local and global procedures. In the local minimization, the sum of the energy terms was minimized locally in the direction in which the total energy gradient was the steepest. In the global energy minimization, a stochastic method was used where random changes, biased towards high-energy regions, were made. Changes which decreased the energy were always retained, whereas changes that increased the energy were kept with a probability that decreased exponentially with increasing energy difference. For each local minimization, we used five times 1000 iterations and, for the global minimization, we used 3000 iterations for each variable that was changed from the original structure.

The energy values obtained were subsequently used as parameters in the prediction algorithm. The energy was minimized on the basis of the following energy terms: electrostatic interactions, hydrogen bonds, van der Waals’ interactions and torsion energy with parameters from the ECEPP/3 force field [28]. The water in the simulation was treated implicitly in order to considerably reduce the simulation time.

For each mutant, the energy value of its corresponding structure was calculated, using the program icm. Furthermore, for each position, the average energy value for 19 structures, representing all possible amino acid residue exchanges, was also calculated. As the central domain of the p53 domain (positions 94–289) contains 196 amino acid residues, a total of 3724 possible mutants was simulated. Each mutant was simulated four times to obtain representative sampling, decreasing the risk of inappropriate energy values as a result of calculations becoming stuck in local minima.

Stability changes on mutation have been investigated previously [29–34] as a complement to other prediction parameters. However, we used a physical effective energy function to calculate the stability changes on mutation, whereas the methods mentioned use either statistical potentials, constructed from atom contact in existing protein structures, or empirical models, based on protein experiments. To speed up calculations, we used an implicit water solvent, which, combined with modern multicore CPUs, makes it possible to simulate all possible mutants in the protein. Yip *et al.* [7] have also used a physical effective energy function simulation, but with a completely different technique, molecular dynamics, compared with our Monte Carlo-based molecular modelling method. They also used a different approach in which they predicted functionally important residues and not the effect of mutations.

Methods have also been developed to predict mutant severity without stability parameters [4,35,36], as have methods that look only at stability changes of the mutation compared with the wild-type protein [22,31].

### Matthews’ correlation coefficient

Matthews’ correlation coefficient (MCC) [37] was used to estimate the performance of the classifications. Values can range from −1 to 1, where 1 is a perfect classification. To obtain a high MCC value, the classification needs to be accurate for both classes. This makes the value unbiased in relation to the difference in class sizes. It also has the effect that it is hard to achieve a high value. For example, a classification with a total accuracy of 85% (90% true positives and 75% true negatives) will only produce an MCC value of 0.66. The MCC value is calculated using the following formula: 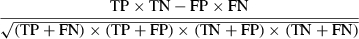


where TP denotes true positives, TN true negatives, FP false positives and FN false negatives.

### Cross-correlation between parameters

To measure the similarity between parameters, we used Pearson’s product-moment correlation coefficient [14] described by the following equation: 
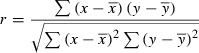


where *x* and *y* are values from the two parameters measured, and 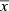 and 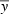 are the mean values for the respective parameters.

### Thermally sensitive mutants

There are several p53 mutants whose activity varies considerably depending on the temperature. Under normal conditions, they have no or very low activity but, if the temperature is lowered by just 7 °C, they behave almost as the wild-type protein. This dataset should be very hard to predict correctly as the stabilities of the mutated proteins are very close to those of the wild-type, yet they have low activity.

## Abbreviations

MCC: Matthews’ correlation coefficient
PLS: partial least squares
ROC: receiver operating characteristic.

## Supporting information

The following supplementary material is available:

**Table S1.** p53 sequences.

**Table S2.** Breast cancer mutations.

This supplementary material can be found in the online version of this article.

Please note: As a service to our authors and readers, this journal provides supporting information supplied by the authors. Such materials are peer-reviewed and may be re-organized for online delivery, but are not copy-edited or typeset. Technical support issues arising from supporting information (other than missing files) should be addressed to the authors.

## References

[1] Sjoblom T, Jones S, Wood LD, Parsons DW, Lin J, Barber TD, Mandelker D, Leary RJ, Ptak J, Silliman N (2006). The consensus coding sequences of human breast and colorectal cancers. Science.

[2] Greenman C, Stephens P, Smith R, Dalgliesh GL, Hunter C, Bignell G, Davies H, Teague J, Butler A, Stevens C (2007). Patterns of somatic mutation in human cancer genomes. Nature.

[3] Chanock SJ, Thomas G (2007). The devil is in the DNA. Nat Genet.

[4] Ng PC, Henikoff S (2001). Predicting deleterious amino acid substitutions. Genome Res.

[5] Ng PC, Henikoff S (2002). Accounting for human polymorphisms predicted to affect protein function. Genome Res.

[6] Tavtigian SV, Deffenbaugh AM, Yin L, Judkins T, Scholl T, Samollow PB, de Silva D, Zharkikh A, Thomas A (2006). Comprehensive statistical study of 452 BRCA1 missense substitutions with classification of eight recurrent substitutions as neutral. J Med Genet.

[7] Yip YL, Zoete V, Scheib H, Michielin O (2006). Structural assessment of single amino acid mutations: application to TP53 function. Hum Mutat.

[8] Soussi T, Beroud C (2001). Assessing TP53 status in human tumours to evaluate clinical outcome. Nat Rev Cancer.

[9] Soussi T, Dehouche K, Beroud C (2000). p53 website and analysis of p53 gene mutations in human cancer: forging a link between epidemiology and carcinogenesis. Hum Mutat.

[10] Kato S, Han SY, Liu W, Otsuka K, Shibata H, Kanamaru R, Ishioka C (2003). Understanding the function–structure and function–mutation relationships of p53 tumor suppressor protein by high-resolution missense mutation analysis. Proc Natl Acad Sci USA.

[11] Greenblatt MS, Beaudet JG, Gump JR, Godin KS, Trombley L, Koh J, Bond JP (2003). Detailed computational study of p53 and p16: using evolutionary sequence analysis and disease-associated mutations to predict the functional consequences of allelic variants. Oncogene.

[12] Robins T, Carlsson J, Sunnerhagen M, Wedell A, Persson B (2006). Molecular model of human CYP21 based on mammalian CYP2C5: structural features correlate with clinical severity of mutations causing congenital adrenal hyperplasia. Mol Endocrinol.

[13] el-Deiry WS, Tokino T, Velculescu VE, Levy DB, Parsons R, Trent JM, Lin D, Mercer WE, Kinzler KW, Vogelstein B (1993). WAF1, a potential mediator of p53 tumor suppression. Cell.

[14] Rodgers JL, Nicewander WA (1988). Thirteen ways to look at the correlation coefficient. Am Stat.

[15] Daffertshofer A, Lamoth CJ, Meijer OG, Beek PJ (2004). PCA in studying coordination and variability: a tutorial. Clin Biomech (Bristol, Avon).

[16] Eriksson L, Johansson E, Kettaneh-Wold N, Wold S (2001). Multi- and Megavariate Data Analysis – Principles and Applications.

[17] Vapnik VN (1975). The Nature of Statistical Learning Theory.

[18] Denissenko MF, Pao A, Tang M, Pfeifer GP (1996). Preferential formation of benzo[a]pyrene adducts at lung cancer mutational hotspots in P53. Science.

[19] Richardson DC, Richardson JS (1992). The kinemage: a tool for scientific communication. Protein Sci.

[20] Mathe E, Olivier M, Kato S, Ishioka C, Hainaut P, Tavtigian SV (2006). Computational approaches for predicting the biological effect of p53 missense mutations: a comparison of three sequence analysis based methods. Nucleic Acids Res.

[21] Dayhoff MO (1978). Atlas of Protein Sequence and Structure.

[22] Parthiban V, Gromiha MM, Schomburg D (2006). CUPSAT: prediction of protein stability upon point mutations. Nucleic Acids Res.

[24] Tom E, Schulman KA (1997). Mathematical models in decision analysis. Infect Control Hosp Epidemiol.

[25] Cho Y, Gorina S, Jeffrey PD, Pavletich NP (1994). Crystal structure of a p53 tumor suppressor–DNA complex: understanding tumorigenic mutations. Science.

[26] Abagyan R, Totrov M (1994). Biased probability Monte Carlo conformational searches and electrostatic calculations for peptides and proteins. J Mol Biol.

[27] Abagyan R, Totrov M, Kuznetsov D (1994). icm– a new method for protein modeling and design: applications to docking and structure prediction from the distorted native conformation. J Comput Chem.

[28] Nemethy G, Gibson KD, Palmer KA, Yoon CN, Paterlini G, Zagari A, Rumsey S, Scheraga HA (1992). Energy parameters in polypeptides. 10. Improved geometrical parameters and nonbonded interactions for use in the ECEPP/3 algorithm, with application to proline-containing peptides. J Phys Chem.

[29] Saqi MA, Goodfellow JM (1990). Free energy changes associated with amino acid substitution in proteins. Protein Eng.

[30] Wang Z, Moult J (2001). SNPs, protein structure, and disease. Hum Mutat.

[31] Guerois R, Nielsen JE, Serrano L (2002). Predicting changes in the stability of proteins and protein complexes: a study of more than 1000 mutations. J Mol Biol.

[32] Capriotti E, Fariselli P, Calabrese R, Casadio R (2005). Predicting protein stability changes from sequences using support vector machines. Bioinformatics.

[33] Feyfant E, Sali A, Fiser A (2007). Modeling mutations in protein structures. Protein Sci.

[34] Barenboim M, Jamison DC, Vaisman II (2005). Statistical geometry approach to the study of functional effects of human nonsynonymous SNPs. Hum Mutat.

[35] Chasman D, Adams RM (2001). Predicting the functional consequences of non-synonymous single nucleotide polymorphisms: structure-based assessment of amino acid variation. J Mol Biol.

[36] Xue D, Yin J, Tan M, Yue J, Wang Y, Liang L (2008). Prediction of functional nonsynonymous single nucleotide polymorphisms in human G-protein-coupled receptors. J Hum Genet.

[37] Matthews BW (1975). Comparison of the predicted and observed secondary structure of T4 phage lysozyme. Biochim Biophys Acta.

[38] Martin AC, Facchiano AM, Cuff AL, Hernandez-Boussard T, Olivier M, Hainaut P, Thornton JM (2002). Integrating mutation data and structural analysis of the TP53 tumor-suppressor protein. Hum Mutat.

[39] Kabsch W, Sander C (1983). Dictionary of protein secondary structure: pattern recognition of hydrogen-bonded and geometrical features. Biopolymers.

[40] Kyte J, Doolittle RF (1982). A simple method for displaying the hydropathic character of a protein. J Mol Biol.

[41] Zimmerman JM, Eliezer N, Simha R (1968). The characterization of amino acid sequences in proteins by statistical methods. J Theor Biol.

[42] Chenna R, Sugawara H, Koike T, Lopez R, Gibson TJ, Higgins DG, Thompson JD (2003). Multiple sequence alignment with the Clustal series of programs. Nucleic Acids Res.

