# Supplementary material for: Investigation and prediction of the severity of p53 mutants using parameters from structural calculations
Source: FEBS J. 2009 Aug;276(15):4142–55. doi: 10.1111/j.1742-4658.2009.07124.x (PMC2730554; doi:10.1111/j.1742-4658.2009.07124.x)
Supplement: Supplementary file 1 [file ejb0276-4142-SD1.pdf]

Table S1 p53 sequences. The species from where the p53 sequences, used in the conservation parameter, were extracted.

Latin	Trivial name	Accession number	Database
Barbus	Barbel	Q9W678	SwissProt
Bos primigenius indicus	Zebu	P67938	SwissProt
Bos taurus	Cow	P67938	SwissProt
Canis	Canis (Wolfs and dogs)	Q29537	SwissProt
Cavia porcellus	Guinea pig	Q9WUR6	SwissProt
Chlorocebus	African Green Monkey	P13481	SwissProt
Cricetulus griseus	Chinese hamster	O09185	SwissProt
Cricetus cricetus	Golden hamster	Q00366	SwissProt
Danio rerio	Zebrafish	P79734	SwissProt
Delphinapterus leucas	Beluga (White whale)	Q8SPZ3	SwissProt
Felis catus	Cat	P41685	SwissProt
Gallus gallus	Chicken	P10360	SwissProt
Homo sapiens	Human	P04637	SwissProt
Ictalurus punctatus	Channel catfish	O93379	SwissProt
Macaca fascicularis	Crab-eating Macaque	P56423	SwissProt
Macaca fuscata	Japanese Macaque	P61260	SwissProt
Macaca mulatta	Rhesus Macaque	P56424	SwissProt
Marmota monax	Woodchuck	O36006	SwissProt
Meriones unguiculates	Gerbil	Q920Y0	SwissProt
Mus musculus	House mouse	P02340	SwissProt
Oncorhynchus mykiss	Rainbow trout	P25035	SwissProt
Oryctolagus cuniculus	Rabbit	Q95330	SwissProt
Ovis aries	Sheep	P51664	SwissProt
Pan troglodytes	Chimpanzee	XP_511957	Refseq
Platichthys flesus	European flounder	O12946	SwissProt
Rattus norvegicus	Rat	P10361	SwissProt
Spalax judaei	Blind mole rats	CAH03844	EMBL
Sus scrofa	Pig	Q9TUB2	SwissProt
Tupaia belangeri	Treeshrew	Q9TTA1	SwissProt
Tetraodon miurus	Congo pufferfish	Q9W679	SwissProt
Xenopus laevis	African clawed frog	P07193	SwissProt
Xenopus tropicalis	Western clawed frog	Q6NTF1	SwissProt
Xiphophorus hellerii	Green swordtail	O57538	SwissProt
Xiphophorus maculates	Southern platyfish	Q92143	SwissProt
