# Supplementary material for: Investigation and prediction of the severity of p53 mutants using parameters from structural calculations
Source: FEBS J. 2009 Aug;276(15):4142–55. doi: 10.1111/j.1742-4658.2009.07124.x (PMC2730554; doi:10.1111/j.1742-4658.2009.07124.x)
Supplement: Supplementary file 2 [file ejb0276-4142-SD2.pdf]

Table S2 Breast cancer mutations

Codon	Wild type amino acid	Mutant amino acid	Measured WAF1 activity	Class WAF1	WAF1 predicted severity	WAF1 predicted class
110	R	C	10.9	2	46.2	2
110	R	L	12.3	2	88.2	2
110	R	P	10.8	2	47.8	2
113	F	C	12.6	2	74.6	2
126	Y	D	12.6	2	83.0	2
127	S	F	12.5	2	117.9	2
128	P	S	48.1	1	33.9	1
129	A	V	67.6	1	25.2	1
130	L	F	9.1	2	58.7	2
130	L	P	10.1	2	164.8	2
130	L	R	8.5	2	44.1	2
130	L	V	8.9	2	59.2	2
131	N	S	10.0	2	55.0	2
132	K	E	0.6	2	80.6	2
132	K	N	10.5	2	86.8	2
132	K	Q	10.9	2	67.7	2
132	K	R	14.1	2	71.0	2
133	M	I	64.4	1	52.7	1
134	F	C	9.5	2	69.7	2
134	F	L	10.7	2	75.4	2
135	C	F	10.4	2	57.1	2
135	C	G	8.3	2	96.1	2
135	C	R	0.9	2	128.2	2
135	C	S	7.4	2	74.1	2
135	C	W	12.8	2	110.6	2
135	C	Y	10.9	2	97.2	2
136	Q	E	12.0	2	69.7	1
136	Q	R	65.5	1	53.5	1
138	A	P	10.9	2	67.6	2
138	A	T	12.3	2	51.7	1
138	A	V	31.8	1	59.4	2
139	K	N	10.4	2	73.5	1
141	C	R	11.0	2	188.1	2
141	C	Y	9.8	2	190.6	2
143	V	A	11.4	2	42.6	2
143	V	M	10.3	2	64.8	2
144	Q	H	16.0	2	50.8	1
144	Q	L	16.5	2	72.8	2
144	Q	P	13.8	2	65.2	2
145	L	P	2.0	2	41.5	2
145	L	Q	12.4	2	38.2	2
145	L	R	12.6	2	149.1	2
146	W	R	58.9	1	31.2	1
147	V	D	11.6	2	76.2	2
149	S	P	21.3	2	82.0	1

Codon	Wild type amino acid	Mutant amino acid	Measured WAF1 activity	Class WAF1	WAF1 predicted severity	WAF1 predicted class
150	T	I	48.7	1	54.1	1
151	P	A	1.5	2	72.2	2
151	P	H	10.8	2	78.7	2
151	P	L	13.2	2	53.9	2
151	P	S	0.9	2	126.3	2
151	P	T	9.4	2	151.8	2
152	P	L	9.5	2	87.5	2
152	P	S	8.5	2	47.8	1
153	P	L	31.8	1	79.7	1
153	P	S	30.0	1	17.3	1
154	G	D	39.7	1	48.4	1
154	G	S	11.5	2	49.5	1
154	G	V	8.4	2	68.2	2
155	T	I	8.9	2	156.6	2
155	T	N	8.6	2	63.1	2
155	T	P	7.8	2	128.4	2
155	T	S	51.8	1	35.2	1
156	R	C	23.9	2	82.9	2
156	R	G	7.5	2	56.5	2
156	R	H	18.6	2	100.0	1
156	R	P	8.2	2	64.4	2
157	V	F	9.1	2	39.8	2
157	V	G	8.8	2	25.6	1
157	V	I	33.6	1	68.5	1
158	R	C	10.1	2	86.0	2
158	R	H	8.8	2	63.9	2
158	R	L	8.2	2	168.4	2
158	R	P	10.2	2	100.1	2
159	A	D	10.2	2	75.3	2
159	A	P	9.7	2	71.2	2
159	A	S	29.7	1	53.7	1
159	A	T	85.3	1	35.2	1
159	A	V	6.9	2	66.4	2
160	M	I	25.3	1	43.3	1
160	M	V	42.2	1	86.6	1
161	A	D	2.4	2	104.7	2
161	A	T	13.3	2	118.7	2
162	I	F	6.5	2	60.1	2
163	Y	C	18.3	2	57.4	2
163	Y	H	11.2	2	59.8	2
163	Y	N	11.4	2	175.2	2
164	K	E	12.4	2	61.4	2
164	K	N	22.3	2	42.0	1
167	Q	L	23.8	2	89.2	1
167	Q	R	37.6	1	23.5	1
168	H	P	12.2	2	45.4	2

Codon	Wild type amino acid	Mutant amino acid	Measured WAF1 activity	Class WAF1	WAF1 predicted severity	WAF1 predicted class
168	H	R	16.4	2	51.2	1
168	H	Y	50.9	1	50.5	1
169	M	I	40.2	1	46.7	1
169	M	V	43.9	1	56.0	1
170	T	A	45.2	1	46.6	1
170	T	S	42.1	1	22.1	1
171	E	G	3.2	2	99.0	2
171	E	K	54.7	1	55.4	1
172	V	D	3.3	2	61.2	2
172	V	F	8.2	2	41.7	2
173	V	A	7.5	2	44.7	2
173	V	L	3.6	2	45.6	2
173	V	M	10.5	2	67.4	2
174	R	K	65.7	1	35.9	1
174	R	W	16.4	2	44.4	2
175	R	G	10.5	2	90.3	2
175	R	H	12.4	2	188.9	2
175	R	L	13.2	2	93.3	2
176	C	F	22.9	2	69.8	2
176	C	G	16.0	2	171.0	2
176	C	R	10.6	2	238.1	2
176	C	S	13.3	2	104.9	2
176	C	W	15.2	2	234.3	2
176	C	Y	14.8	2	185.6	2
177	P	R	17.9	2	80.1	2
177	P	S	34.3	1	47.0	1
178	H	D	13.4	2	46.3	1
178	H	P	14.1	2	92.0	2
178	H	R	35.9	1	31.9	1
179	H	D	21.6	2	86.0	2
179	H	L	21.3	2	58.6	2
179	H	N	19.3	2	77.9	2
179	H	Q	17.5	2	169.8	2
179	H	R	13.0	2	153.5	2
179	H	Y	13.3	2	107.4	2
181	R	C	26.1	1	57.5	2
181	R	H	34.1	1	34.6	1
181	R	P	14.1	2	53.1	1
184	D	N	56.1	1	64.1	1
185	S	R	69.4	1	44.0	1
189	A	V	56.1	1	50.8	1
190	P	L	10.1	2	47.3	2
191	P	L	25.7	1	108.7	1
192	Q	R	31.6	1	26.8	1
193	H	D	11.7	2	76.6	2
193	H	L	11.0	2	87.8	2

Codon	Wild type amino acid	Mutant amino acid	Measured WAF1 activity	Class WAF1	WAF1 predicted severity	WAF1 predicted class
193	H	P	10.0	2	105.5	2
193	H	R	10.2	2	67.4	2
193	H	Y	5.0	2	38.2	2
194	L	F	14.4	2	60.4	2
194	L	P	0.3	2	92.1	2
194	L	R	10.6	2	77.4	2
195	I	F	10.5	2	54.5	1
195	I	T	11.2	2	62.9	2
196	R	P	12.2	2	88.4	2
196	R	Q	24.6	2	82.6	2
197	V	G	7.9	2	60.7	2
197	V	L	12.4	2	54.6	2
198	E	K	66.2	1	110.8	1
199	G	R	45.8	1	34.3	1
202	R	H	49.8	1	31.9	1
202	R	S	47.6	1	111.4	1
205	Y	C	8.9	2	84.7	2
205	Y	D	9.6	2	70.7	2
205	Y	S	8.9	2	101.2	2
208	D	E	11.6	2	57.8	2
208	D	V	4.7	2	63.4	2
209	R	K	112.7	1	21.3	1
211	T	A	5.9	2	31.9	1
212	F	L	70.4	1	17.9	1
213	R	L	0.7	2	55.7	2
213	R	Q	2.2	2	69.1	2
214	H	R	3.1	2	57.6	2
214	H	Y	3.2	2	57.1	2
215	S	C	25.5	1	50.5	2
215	S	G	2.7	2	63.9	2
215	S	I	8.1	2	69.6	2
215	S	N	4.0	2	129.2	2
216	V	E	17.7	2	70.8	2
216	V	L	2.7	2	46.4	2
216	V	M	0.2	2	129.3	2
217	V	M	95.1	1	85.9	1
218	V	A	69.3	1	82.3	2
218	V	E	4.4	2	56.0	2
218	V	M	0.8	2	50.7	1
219	P	L	4.2	2	99.5	2
220	Y	C	1.2	2	91.0	2
220	Y	H	1.0	2	42.1	2
220	Y	S	0.0	2	84.9	2
221	E	D	126.0	1	47.7	1
222	P	S	82.9	1	38.0	1
224	E	D	59.7	1	68.4	1

Codon	Wild type amino acid	Mutant amino acid	Measured WAF1 activity	Class WAF1	WAF1 predicted severity	WAF1 predicted class
224	E	K	38.5	1	42.3	1
226	G	D	72.7	1	20.5	1
227	S	F	21.8	2	73.4	2
228	D	G	75.4	1	38.7	1
228	D	N	22.8	2	42.0	1
230	T	I	52.3	1	154.3	2
232	I	F	2.5	2	51.9	1
232	I	S	0.0	2	39.1	2
232	I	T	1.9	2	70.2	2
233	H	Y	77.2	1	43.7	1
234	Y	C	2.1	2	173.9	2
234	Y	H	0.0	2	116.1	2
234	Y	N	0.4	2	67.9	2
235	N	S	73.1	1	108.8	1
236	Y	C	0.7	2	191.0	2
236	Y	N	80.2	1	68.6	2
237	M	I	0.4	2	41.3	2
237	M	K	14.2	2	192.2	2
237	M	V	13.8	2	107.3	1
238	C	F	0.8	2	65.2	2
238	C	G	13.9	2	90.6	2
238	C	R	0.5	2	188.5	2
238	C	S	15.2	2	104.2	2
238	C	Y	14.6	2	159.5	2
239	N	D	20.2	2	97.6	2
239	N	K	18.1	2	150.0	2
239	N	S	14.9	2	166.7	2
239	N	T	1.1	2	115.2	2
240	S	I	6.0	2	65.8	2
240	S	R	12.2	2	163.0	2
241	S	A	15.7	2	78.3	2
241	S	C	0.0	2	90.8	2
241	S	F	0.0	2	75.3	2
241	S	P	0.5	2	45.4	2
242	C	F	13.8	2	59.8	2
242	C	G	0.0	2	58.3	2
242	C	S	0.0	2	160.1	2
242	C	W	14.2	2	55.3	2
242	C	Y	0.0	2	179.0	2
243	M	I	46.4	1	126.7	2
243	M	L	0.0	2	110.4	1
243	M	T	7.2	2	160.9	2
244	G	C	0.0	2	56.3	1
244	G	D	0.5	2	46.1	1
244	G	S	0.3	2	29.5	1
244	G	V	0.0	2	70.8	2

Codon	Wild type amino acid	Mutant amino acid	Measured WAF1 activity	Class WAF1	WAF1 predicted severity	WAF1 predicted class
245	G	C	0.0	2	61.5	2
245	G	D	2.0	2	78.3	2
245	G	R	7.9	2	128.5	2
245	G	S	0.0	2	80.0	2
245	G	V	0.0	2	96.3	2
246	M	I	0.3	2	38.0	2
246	M	L	0.0	2	63.5	2
246	M	R	2.9	2	175.4	2
246	M	T	0.0	2	112.6	2
246	M	V	0.0	2	134.7	2
248	R	G	0.0	2	154.8	2
248	R	L	0.0	2	164.4	2
248	R	P	6.5	2	199.3	2
248	R	Q	0.0	2	44.2	2
248	R	W	0.0	2	175.6	2
249	R	G	0.2	2	94.2	2
249	R	K	1.2	2	76.2	2
249	R	M	0.0	2	94.9	2
249	R	S	12.4	2	85.7	2
249	R	T	0.0	2	126.1	2
249	R	W	6.8	2	110.4	2
250	P	A	46.6	1	34.4	1
250	P	L	0.0	2	68.9	2
250	P	S	36.8	1	110.0	1
251	I	N	8.8	2	89.3	2
251	I	S	0.0	2	66.3	2
251	I	V	11.9	2	116.4	2
252	L	F	63.2	1	66.3	1
253	T	I	4.1	2	81.8	2
254	I	N	6.2	2	44.8	2
254	I	T	0.9	2	70.6	2
255	I	F	0.5	2	115.0	2
255	I	N	7.0	2	68.0	2
255	I	T	11.9	2	69.5	2
256	T	A	10.8	2	97.0	1
256	T	I	5.8	2	83.6	2
257	L	P	12.6	2	53.3	2
258	E	G	0.0	2	45.9	2
258	E	K	0.3	2	67.5	2
258	E	Q	16.4	2	112.7	2
259	D	V	10.9	2	68.6	1
259	D	Y	9.9	2	61.4	2
262	G	S	5.8	2	44.5	1
262	G	V	11.7	2	36.7	2
263	N	D	18.1	2	24.5	1
265	L	P	0.0	2	185.5	2

Codon	Wild type amino acid	Mutant amino acid	Measured WAF1 activity	Class WAF1	WAF1 predicted severity	WAF1 predicted class
266	G	E	0.0	2	82.5	2
266	G	R	10.8	2	80.2	2
266	G	V	0.0	2	113.5	2
267	R	P	0.0	2	76.4	2
267	R	Q	9.8	2	41.3	2
269	S	N	17.1	2	60.1	2
270	F	C	0.0	2	71.3	2
270	F	L	8.1	2	60.5	2
270	F	S	0.0	2	49.9	2
270	F	Y	62.7	1	57.3	1
271	E	K	8.6	2	151.0	2
271	E	Q	13.0	2	82.5	2
272	V	A	1.0	2	125.4	2
272	V	G	0.0	2	120.0	2
272	V	L	7.1	2	43.0	2
272	V	M	8.8	2	66.1	2
273	R	C	0.9	2	183.1	2
273	R	G	16.7	2	156.8	2
273	R	H	1.0	2	85.2	2
273	R	L	0.9	2	171.0	2
273	R	P	0.6	2	163.0	2
273	R	S	17.4	2	63.4	2
274	V	A	1.4	2	117.8	2
274	V	D	1.3	2	37.4	2
274	V	G	5.3	2	115.7	2
274	V	I	119.1	1	69.4	1
275	C	F	0.0	2	119.2	2
275	C	G	0.6	2	91.4	2
275	C	R	0.4	2	139.1	2
275	C	S	0.6	2	187.5	2
275	C	W	0.7	2	130.4	2
275	C	Y	0.4	2	168.5	2
276	A	D	0.4	2	58.7	2
276	A	P	0.6	2	51.4	1
276	A	T	20.2	2	26.6	1
276	A	V	10.5	2	24.4	1
277	C	F	0.3	2	150.4	2
277	C	Y	0.7	2	161.5	2
278	P	A	14.9	2	46.6	2
278	P	H	0.3	2	156.6	2
278	P	L	0.8	2	87.4	2
278	P	R	0.7	2	79.5	2
278	P	S	0.3	2	124.2	2
278	P	T	0.1	2	67.7	2
279	G	E	0.3	2	68.4	2
280	R	G	21.1	2	142.6	2

Codon	Wild type amino acid	Mutant amino acid	Measured WAF1 activity	Class WAF1	WAF1 predicted severity	WAF1 predicted class
280	R	I	0.2	2	117.6	2
280	R	K	0.5	2	65.4	2
280	R	T	0.3	2	114.7	2
281	D	E	1.7	2	89.9	2
281	D	G	12.1	2	61.1	2
281	D	H	0.7	2	73.6	2
281	D	N	0.5	2	84.4	2
282	R	G	0.4	2	167.9	2
282	R	L	28.0	1	112.1	2
282	R	P	0.0	2	92.1	2
282	R	Q	7.2	2	75.5	2
282	R	W	0.6	2	108.5	2
283	R	C	25.3	1	158.3	2
283	R	H	0.5	2	133.3	2
283	R	P	0.2	2	155.8	2
284	T	P	7.3	2	83.6	2
285	E	K	0.6	2	41.5	2
286	E	A	0.2	2	46.8	2
286	E	G	0.1	2	77.2	2
286	E	K	11.1	2	71.1	2
287	E	K	56.7	1	33.4	1
